# EBV-miR-BART10-3p facilitates epithelial-mesenchymal transition and promotes metastasis of nasopharyngeal carcinoma by targeting BTRC

**DOI:** 10.18632/oncotarget.6155

**Published:** 2015-10-19

**Authors:** Qijia Yan, Zhaoyang Zeng, Zhaojian Gong, Wenling Zhang, Xiayu Li, Baoyu He, Yali Song, Qiao Li, Yong Zeng, Qianjin Liao, Pan Chen, Lei Shi, Songqing Fan, Bo Xiang, Jian Ma, Ming Zhou, Xiaoling Li, Jianbo Yang, Wei Xiong, Guiyuan Li

**Affiliations:** ^1^ Hunan Key Laboratory of Translational Radiation Oncology, Hunan Cancer Hospital and The Affiliated Cancer Hospital of Xiangya School of Medicine, Central South University, Changsha, Hunan, China; ^2^ The Key Laboratory of Carcinogenesis of the Chinese Ministry of Health and The Key Laboratory of Carcinogenesis and Cancer Invasion of the Chinese Ministry of Education, Cancer Research Institute, Central South University, Changsha, Hunan, China; ^3^ Hunan Key Laboratory of Nonresolving Inflammation and Cancer, Disease Genome Research Center, The Third Xiangya Hospital, Central South University, Changsha, Hunan, China; ^4^ The Second Xiangya Hospital, Central South University, Changsha, Hunan, China; ^5^ Department of Laboratory Medicine and Pathology and Masonic Cancer Center, University of Minnesota, Minneapolis, Minnesota, United States of America

**Keywords:** Epstein-Barr virus (EBV), nasopharyngeal carcinoma (NPC), EBV-miR-BART10-3p, BTRC, epithelial-mesenchymal transition (EMT)

## Abstract

Epstein-Barr virus (EBV) infection is closely associated with tumorigenesis and development of nasopharyngeal carcinoma (NPC), but the underlying molecular mechanisms remain poorly understood. It has been recently reported that EBV encodes 44 mature miRNAs, some of which were found to promote tumor development by targeting virus-infected host genes or self-viral genes. However, few targets of EBV encoded-miRNAs that are related to NPC development have been identified to date. In this study, we revealed that in NPC cells, EBV-miR-BART10-3p directly targets *BTRC* gene that encodes βTrCP (beta-transducin repeat containing E3 ubiquitin protein ligase). We found that EBV-miR-BART10-3p expression in clinical samples from a cohort of 106 NPC patients negatively correlated with *BTRC* expression levels. Over-expression of EBV-miR-BART10-3p and down-regulation of *BTRC* were associated with poor prognosis in NPC patients. EBV-miR-BART10-3p promoted the invasion and migration cabilities of NPC cells through the targeting of *BTRC* and regulation of the expression of the downstream substrates β-catenin and Snail. As a result, EBV-miR-BART10-3p facilitated epithelial-mesenchymal transition of NPC. Our study presents an unreported mechanism underlying EBV infection in NPC carcinogenesis, and provides a potential novel biomarker for NPC diagnosis, treatment and prognosis.

## INTRODUCTION

Nasopharyngeal carcinoma (NPC) is a rare type of head and neck cancer in most parts of the world, but has a notably high prevalence in southern China. Recent studies have shown that NPC is closely associated with environmental and genetic factors [1–4]. Among these factors, Epstein-Barr virus (EBV) is one environmental carcinogen related to NPC [5–8]. EBV is a ubiquitous human herpesvirus in which latent infection is associated with malignancies including NPC [5–8], gastric carcinoma [9], and multiple types of B-cell lymphomas [10–12]. Although radiotherapy has been shown to be an effective treatment for NPC patients in early-stages of the disease, the majority (75-90%) of NPC cases are predisposed to metastasis at initial diagnosis [13], which hampers efficacious treatment and poses a high risk of disease recurrence. Better understanding of the mechanisms by which EBV alters nasopharyngeal cells may provide more rational therapeutic targets for NPC.

It has been reported that EBV encodes 44 mature miRNAs that are grouped in two clusters located around the BHRF1 gene and within the BART transcript [14–16]. Some EBV miRNAs target their own viral genes, such as LMP1 [17] and EBNA2 [18], that produce oncogenic proteins of EBV. Moreover, EBV miRNAs are also involved in the regulation of multiple cellular responses, such as cell proliferation, cell-cycle progression, apoptosis and metastasis by targeting virus-infected host genes [19–21]. These findings suggest that EBV miRNAs might exert a variety of important regulatory functions in tumorigenesis and progression of NPC. The function of most EBV-encoded miRNAs remains to be elucidated. In our previous study, we have performed miRNA profiling for all 44 EBV-encoded-miRNAs, using 16 NPC biopsies and 5 non-cancerous nasopharyngeal tissues. Our study found that most EBV miRNAs located in the BART region were highly expressed in NPC samples [22], consistent with previous studies [23, 24]. Through bioinformatic analysis of the regulatory network of EBV miRNAs and host genes, we found that the *BTRC* gene was predicted as a target of multiple EBV encoded miRNAs. It encodes an important component of SCF (Skp1-Cullin1-F-box) E3 ubiquitin ligase, also known as βTrCP (beta-transducin repeat containing E3 ubiquitin protein ligase). Our previous microarray data showed that a decrease in *BTRC* expression was found in NPC samples [25, 26], suggesting that EBV miRNAs might regulate NPC development through its host gene *BTRC*. However, the mechanism by which EBV miRNAs regulate *BTRC* expression and the biological function of *BTRC* in NPC is still largely unknown at present.

To this end, we investigated the effect of EBV-miR-BART10-3p on *BTRC* expression in NPC cells. Meanwhile, we examined the correlation of EBV-miR-BART10-3p with *BTRC* expression and their association with the prognosis of NPC patients. To elucidate the mechanism underlying the function of EBV-miR-BART10-3p in NPC, we also examined the effect of EBV-miR-BART10-3p on invasion and migration of NPC cells and evaluated its potential in regulation of the epithelial-mesenchymal transition (EMT) by regulating EMT-related genes, such as β-catenin and Snail that are downstream substrates of *BTRC*.

## RESULTS

### Highly expressed EBV-miR-BART10-3p was associated with poor survival of NPC patients and inversely correlated to *BTRC* expression in NPC samples

**Figure 1:**
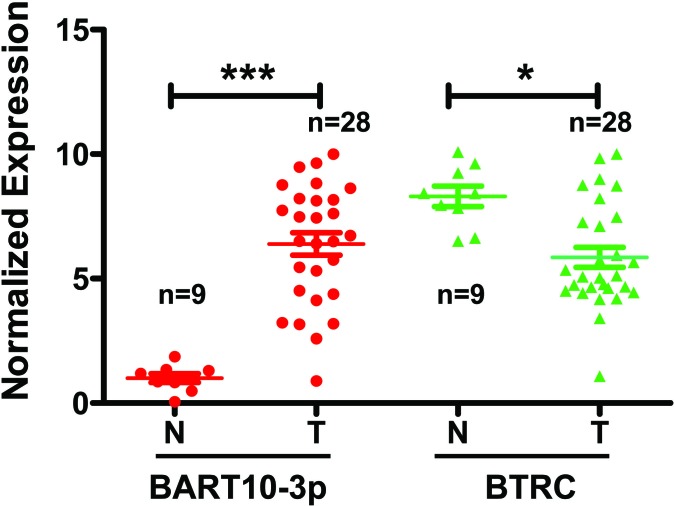
The correlation between the expression of *BTRC* mRNA and EBV-miR-BART10-3p was analyzed by real-time PCR data obtained from 28 NPC tissues and 9 non-tumor nasopharyngeal epithelial tissues N, non-tumor nasopharyngeal epitheliums; T, NPC. N, *n* = 9; T, *n* = 28, *, *p* < 0.05; ***, *p* < 0.001).

**Figure 2:**
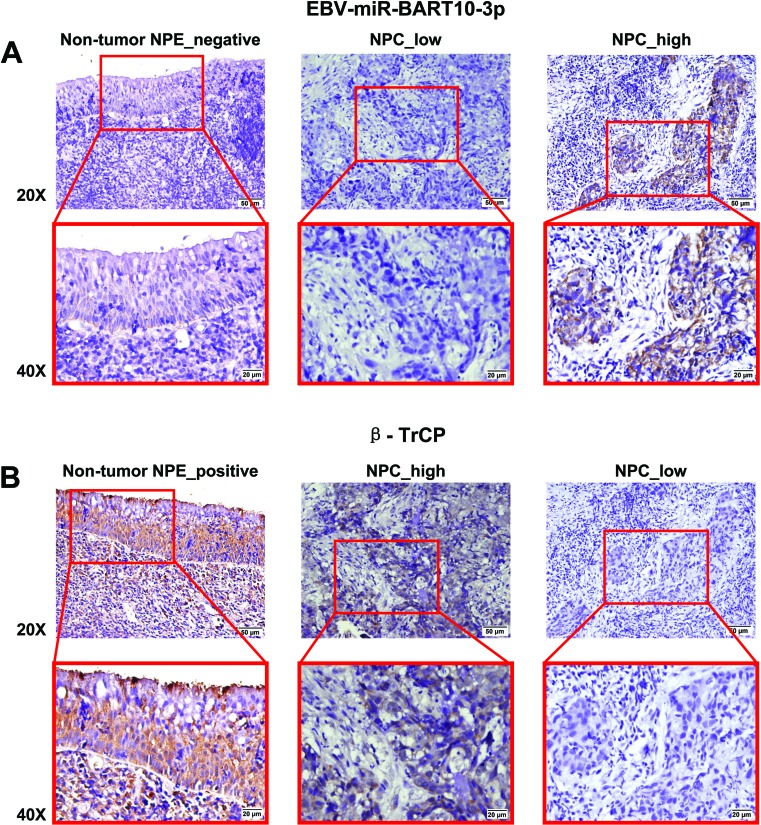

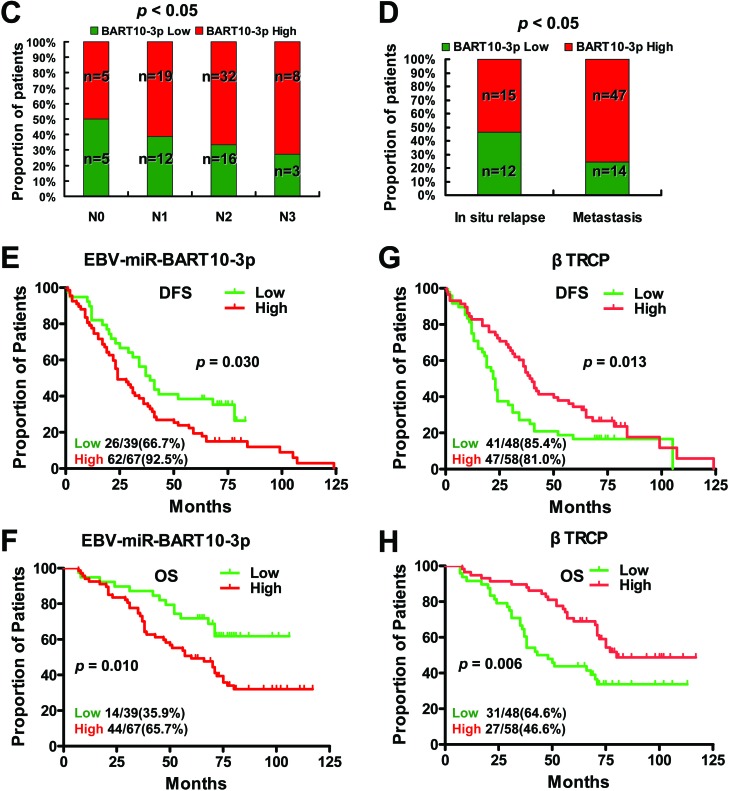
The inverse correlation between high expression of EBV-miR-BART10-3p and low expression of βTrCP in NPC and their expression was associated with poor survival of NPC patients **A.** Comparison of the expression of EBV-miR-BART10-3p between 106 NPC tissue samples and adjacent epithelial tissues was performed by *in situ* hybridization (ISH). As shown in representative images, high expression of EBV-miR-BART10-3p was detected in NPC tissues, as compared to adjacent epithelial tissues. **B.** βTrCP expression was inversely correlated with EBV-miR-BART10-3p in the same cohort of NPC tissues and adjacent epithelial tissues, detected by immunohistochemistry (IHC). **C.** Overexpression of EBV-miR-BART10-3p in NPC was associated with lymph-vascular invasion (*p* < 0.05). **D.** The highly expressed EBV-miR-BART10-3p was correlated with *in situ* relapse (*n* = 27) or distant metastasis (n = 61) in NPC patients (*p* < 0.05). **E.** and **F.** The highly expressed EBV-miR-BART10-3p was correlated with shorter disease free survival (DFS, *p* = 0.030, E) or overall survival (OS, *p* = 0.010, F) of NPC patients. **G.** and **H.** The low expressed βTrCP expression was correlated with shorter disease free survival (DFS, *p* = 0.013, G) or overall survival (OS, *p* = 0.006, H) of NPC patients.

In this study, we first examined the expression of both EBV-miR-BART10-3p and *BTRC* mRNA in 28 NPC and 9 non-tumor nasopharyngeal epithelial biopsies by real-time PCR. We found that EBV-miR-BART10-3p was highly expressed in these clinical samples of NPC, while *BTRC* was expressed at a low level, with expression negatively correlating with EBV-miR-BART10-3p expression (Figure 1). Furthermore, the expression levels of EBV-miR-BART10-3p and βTrCP protein, which is encoded by *BTRC* gene, were evaluated by *in situ* hybridization (ISH) and immunohistochemistry (IHC), respectively, in 106 archived paraffin embedded biopsies. Results showed that EBV-miR-BART10-3p was highly expressed in NPC tissues, as compared to adjacent non-tumor nasopharyngeal epithelial (NPE) tissues (Figure 2A), but βTrCP expression was expressed at low levels in NPC (Figure 2B). We also analyzed the correlation of both EBV-miR-BART10-3p and βTrCP expression with clinicopathological parameters, such as gender, age, histological type, pathological stage, tumor size (T stage), lymph-vascular invasion (N stage) and relapse. Our data found that in these NPC samples, EBV-miR-BART10-3p expression was positively associated with N stage (Figure 2C) and distant tumor metastasis (Figure 2D, Supplemental Table S1). The correlation of EBV-miR-BART10-3p or βTrCP expression with relapse or cancer-related deaths was examined using a Kaplan-Meier survival analysis. The overexpression of EBV-miR-BART10-3p in NPC patients was significantly associated with poor disease-free survival (DFS) and overall survival (OS) (*p* = 0.030 and 0.010, respectively, Figure 2E and Figure 2F) and that the low expression levels of βTrCP in NPC patients was significantly associated with poor DFS and OS (*p* = 0.013 and 0.006, respectively, Figure 2G and Figure 2H). These results strongly suggested that aberrant expression of EBV-miR-BART10-3p and βTrCP might be involved in the progression and metastasis of NPC.

### EBV-miR-BART10-3p targeted *BTRC* gene and inhibited its expression in NPC cells

**Figure 3:**
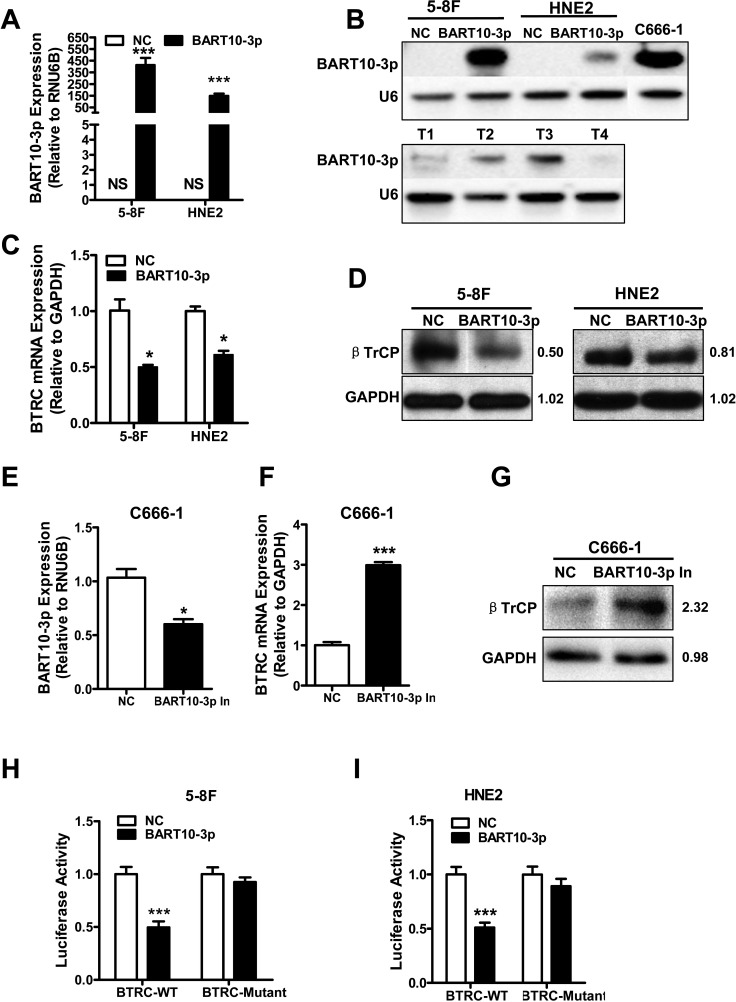
EBV-miR-BART10-3p targeted *BTRC* gene and inhibited its expression in NPC cells EBV negative NPC cell lines HNE2 and 5-8F were transfected by EBV-miR-BART10-3p mimics (BART10-3p) or negative control (NC) respectively. Expression of exogenous BART10-3p was detected by real-time PCR **A.** or Northern blotting **B.**. NS: no signaling. C666-1 was served as positive control in Northern blotting, T1, T2, T3 and T4 are four NPC primary biopsies, the U6 RNA probe was used as an internal loading control. The expression of *BTRC* at the mRNA **C.** or protein **D.** levels were decreased in the EBV-miR-BART10-3p mimics transfected NPC cells, as compared to one with negative control (NC), detected by real-time PCR or western blotting. **E.** EBV-miR-BART10-3p expression was significantly inhibited by a synthesized inhibitor (BART10-3p In) in C666-1, a EBV-positive NPC cell line, as compared to negative control (NC) one. The mRNA **F.** and protein **G.** expression levels of *BTRC* were increased in C666-1 cells transfected with EBV-miR-BART10-3p inhibitor. *BTRC* as a direct target of EBV-miR-BART10-3p was confirmed in HNE2 **H.** and 5-8F **I.** cells by co-transfection with EBV-miR-BART10-3p mimics and luciferase reporter containing either wild type (*BTRC*-WT) or mutated (*BTRC*-mutant) EBV-miR-BART10-3p binding sites in *BTRC* 3′-UTR. EBV-miR-BART10-3p mimics attenuated the luciferase activity of *BTRC*-WT, rather than *BTRC*-mutant (*, *p* < 0.05; ***, *p* < 0.001, Figures are representative of three experiments).

According to bioinformatics analysis, we predicted that the *BTRC* gene might be regulated by multiple EBV encoded miRNAs, such as BART4, BART4*, BART6-3p, BART10-3p, BART18-5p, and BART19-5p [22]. To verify this prediction, we firstly examined the effects of these EBV miRNAs on *BTRC* expression. The results showed that only EBV-miR-BART10-3p could significantly inhibit *BTRC* expression, rather than other EBV miRNAs (data not shown). EBV-miR-BART10-3p mimics was transfected into two EBV negative NPC cell lines, HNE2 and 5-8F, which confirmed by Real-time PCR (Figure 3A) and Northern blotting (Figure 3B), that the expression of *BTRC* was significantly decreased at both the mRNA (Figure 3C) and protein (Figure 3D) levels. Whereas in EBV positive NPC cell line C666-1, the inhibition of endogenous EBV-miR-BART10-3p (Figure 3E) induced *BTRC* expression at both the mRNA (Figure 3F) and protein levels (Figure 3G). To elucidate *BTRC* as a direct target of EBV-miR-BART10-3p, two luciferase reporter vectors were established, which had either wild type (WT) binding sequence of EBV-miR-BART10-3p (*BTRC*-WT) or mutant in the region of *BTRC* 3′-UTR (*BTRC*-mutant). Direct targeting of EBV-miR-BART10-3p to the region of *BTRC* 3′-UTR was confirmed by co-transfection of EBV-miR-BART10-3p mimics and the constructed luciferase reporter vector in HNE2 or 5-8F cells. EBV-miR-BART10-3p significantly attenuated the luciferase activity of *BTRC*-WT, but no effect on *BTRC*-mutant (Figure 3H & 3I). Above all, the results suggested that EBV-miR-BART10-3p could inhibit *BTRC* expression in NPC cells through binding to the specific sites within the 3′-UTR of *BTRC* gene and inhibit its translation.

### EBV-miR-BART10-3p promoted invasion and migration of NPC cells by reducing *BTRC* expression

**Figure 4:**
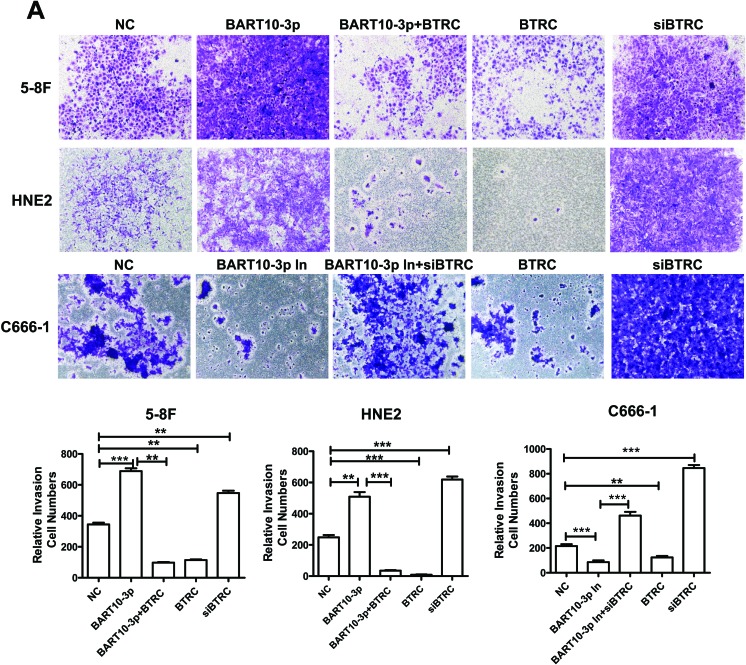

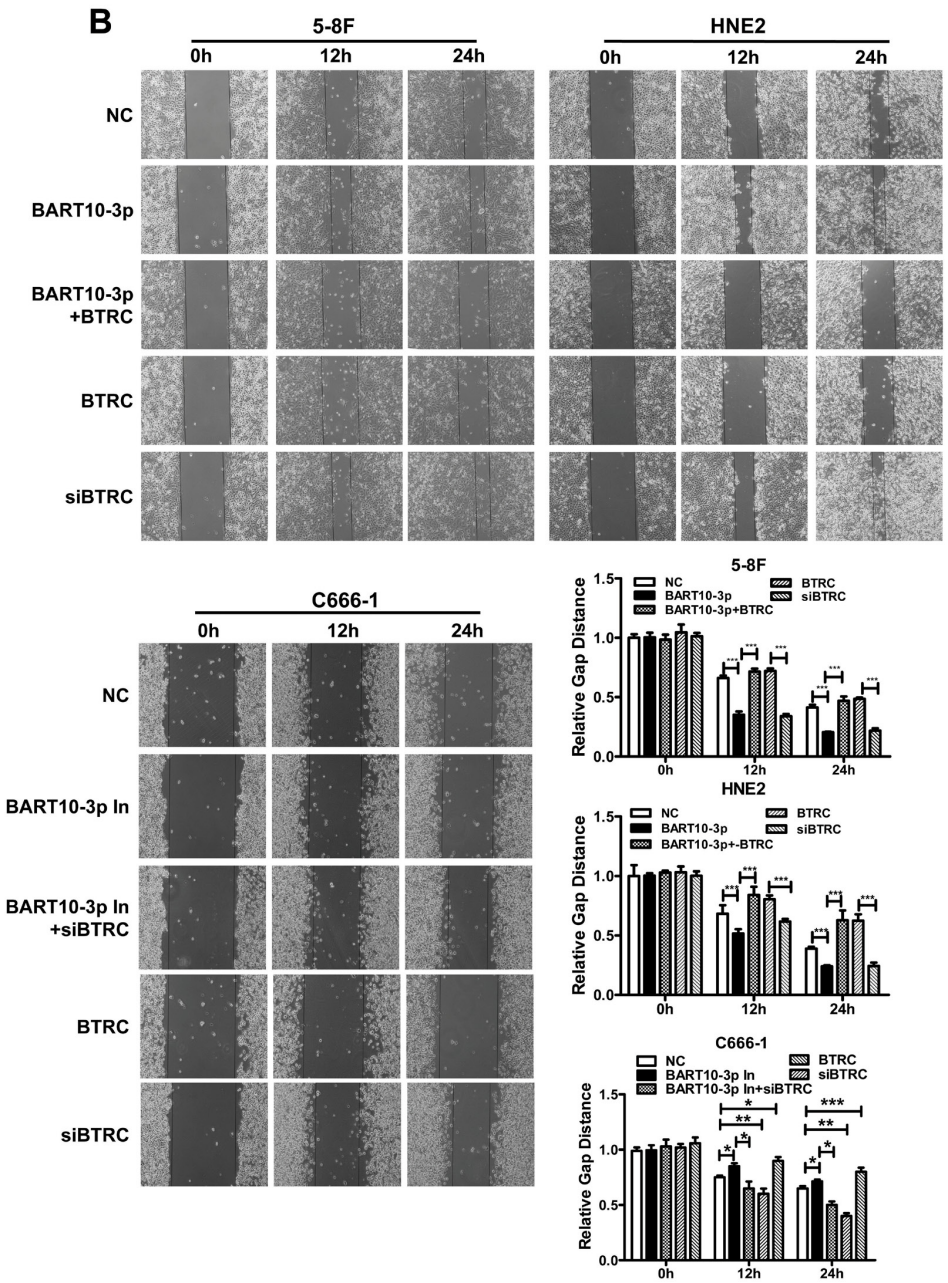
EBV-miR-BART10-3p promoted invasion and migration of NPC cells by reducing *BTRC* expression **A.** The invasion ability was evaluated by transwell assay in EBV negative NPC cells HNE2 and 5-8F or EBV positive cells C666-1. EBV-miR-BART10-3p mimics (BART10-3p), *BTRC* overexpression vector (*BTRC*), BART10-3p+*BTRC* or *BTRC* siRNA were transfected into HNE2 and 5-8F cells, respectively. EBV-miR-BART10-3p inhibitors (BART10-3p In), *BTRC* overexpression vector (*BTRC*), *BTRC* siRNA (si*BTRC*), or BART10-3p In+si*BTRC* were transfected into C666-1 cells, respectively. EBV-miR-BART10-3p mimics could significantly promote invasion of NPC cells, whereas the EBV-miR-BART10-3p mimics-enhanced tumor cell invasion and migration were rescued by overexpression of *BTRC*. **B.** Wound healing assay showed that both EBV-miR-BART10-3p mimics and *BTRC* siRNA accelerated would gap closure, as compared with those with negative control (NC). Overexpression of *BTRC* in HNE2 and 5-8F cells reduced the migration ability, leading to a delayed wound gap closure. Wound healing assay were also performed after EBV-miR-BART10-3p inhibitors (BART10-3p In), *BTRC* overexpression vector (*BTRC*), *BTRC* siRNA (si*BTRC*), or BART10-3p In+si*BTRC* transfection in C666-1 cells. The migration ability could be blocked by BART10-3p In or *BTRC* expression vector. The BART10-3p In-blocked migration ability of C666-1 cells was rescued by si*BTRC*, and si*BTRC* alone also increased the migration ability. The cells in five randomly selected fields were counted and the data were shown as the mean ± SD (*, *p* < 0.05; **, *p* < 0.01; ***, *p* < 0.001).

To further investigate the function of EBV-miR-BART10-3p in NPC cells, HNE2 and 5-8F cells were transfected with EBV-miR-BART10-3p mimics, *BTRC* over-expression vector or *BTRC* siRNA. The abilities of invasion and migration of those transfected cells were measured using transwell and wound healing assays. Results showed that EBV-miR-BART10-3p mimics could significantly promote invasion (Figure 4A) and migration (Figure 4B) of EBV negative NPC cells. The similar effect was observed in *BTRC* siRNA-transfected cells. On the other hand, the EBV-miR-BART10-3p mimics-enhanced tumor cell invasion and migration were rescued by overexpression of *BTRC* in either HNE2 or 5-8F cells. Then we examined whether EBV-miR-BART10-3p inhibitors had the opposite function of EBV-miR-BART10-3p mimics and depressed cell invasion and migration in EBV positive cell line C666-1. Transwell and wound healing assays showed that EBV-miR-BART10-3p inhibitors reduced the abilities of invasion and migration of C666-1 cells (Figure 4). The invasion and migration capacity also decreased in overexpressed-*BTRC* C666-1 cells, which was similar to HNE2 and 5-8F cells. However, *BTRC* siRNA increased this ability and reversed the function of EBV-miR-BART10-3p inhibitors when *BTRC* siRNA and EBV-miR-BART10-3p inhibitors were co-transfected into C666-1 cells. These results suggest that EBV-miR-BART10-3p promotes the abilities of invasion and migration of NPC cells by targeting its target gene *BTRC*.

### EBV-miR-BART10-3p up-regulated the expression of β-catenin and Snail by suppressing *BTRC*

**Figure 5:**
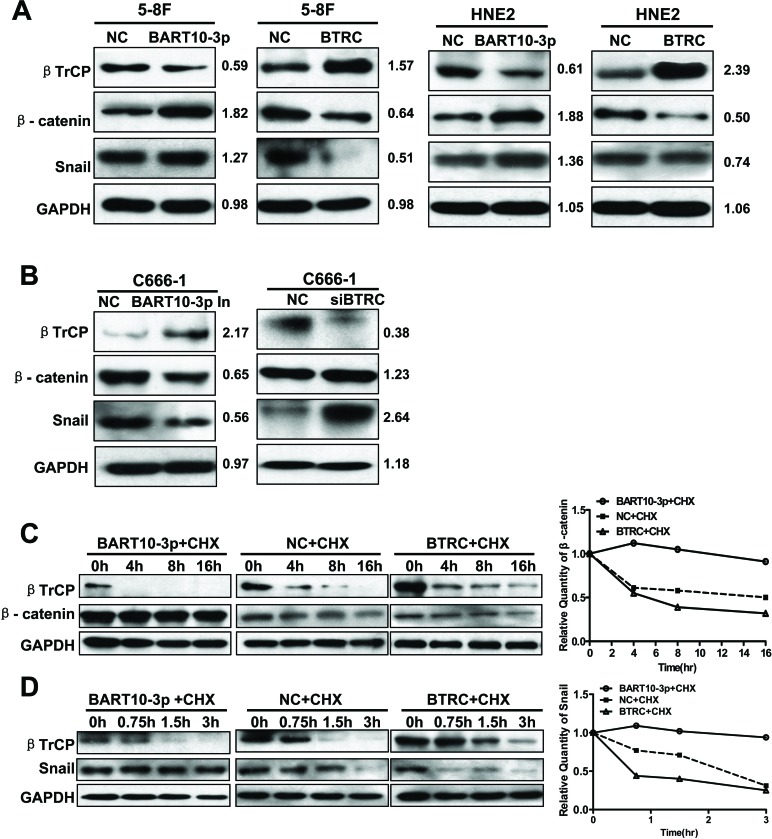
EBV-miR-BART10-3p up-regulated the expression of β-catenin and Snail through inhibiting βTrCP **A.** Western blot analysis of the expression of *BTRC* and its substrates β-catenin and Snail in EBV negative NPC cell lines HNE2 and 5-8F transfected with EBV-miR-BART10-3p mimics (BART10-3p) or *BTRC* expression vector (*BTRC*). **B.** Western blot analysis of the expression of *BTRC* and its substrates β-catenin and Snail in EBV positive NPC cell lines C666-1 transfected with EBV-miR-BART10-3p inhibitors (BART10-3p In) or *BTRC* siRNA (si*BTRC*). **C.** and **D.** The effect of EBV-miR-BART10-3p mimics (BART10-3p) or *BTRC* expression vector (*BTRC*) on ubiquitination degradation rate of β-catenin **C.** and Snail **D.** in 5-8F cells was detected by western blotting at the indicated time point after treatment with Cycloheximide (CHX), an inhibitor of protein biosynthesis. GAPDH was used as an internal loading control.

It has been reported that β-catenin [27] and Snail [28] are substrates of βTrCP. Therefore, we next examined whether EBV-miR-BART10-3p in NPC cells could regulate the expression of β-catenin and Snail by targeting *BTRC* gene. The results revealed that the expression of β-catenin and Snail was significantly enhanced with a decrease of βTrCP expression after EBV-miR-BART10-3p mimics transfection in EBV negative NPC HNE2 and 5-8F cell lines (Figure 5A). Conversely, transfection of EBV-miR-BART10-3p inhibitor in EBV positive NPC C666-1 cell line increased the expression of βTrCP, leading to downregulation of β-catenin and Snail expression (Figure 5B). The inhibitory effect of *BTRC* on β-catenin and Snail was also confirmed by overexpression of *BTRC* in EBV negative cell lines 5-8F and HNE2 (Figure 5A) or knockdown the expression of *BTRC* in EBV positive cell line C666-1 (Figure 5B). Given that β-catenin and Snail were the degradation substrates of βTrCP, we further explored whether up-regulation of β-catenin and Snail expression by EBV-miR-BART10-3p mimics was owing to the inhibition of βTrCP E3 ubiquitin ligase activity. To confirm this hypothesis, cycloheximide (CHX) was added into NPC cells transfected with EBV-miR-BART10-3p mimics or *BTRC* expression vector. The degradation rate of β-catenin and Snail in the transfected cells was observed in different time courses. The results showed that the degradation of β-catenin (Figure 5C) and Snail (Figure 5D) protein was much slower in EBV-miR-BART10-3p mimics-transfected cells than that in the cells transfected with ectopic expressed *BTRC* or negative control. These results indicated that the accumulation of β-catenin and Snail expression in NPC cells by EBV-miR-BART10-3p depended on its inhibition of β*TrCP*-mediated ubiquitination.

### EBV-miR-BART10-3p facilitated the EMT of NPC cells

**Figure 6:**
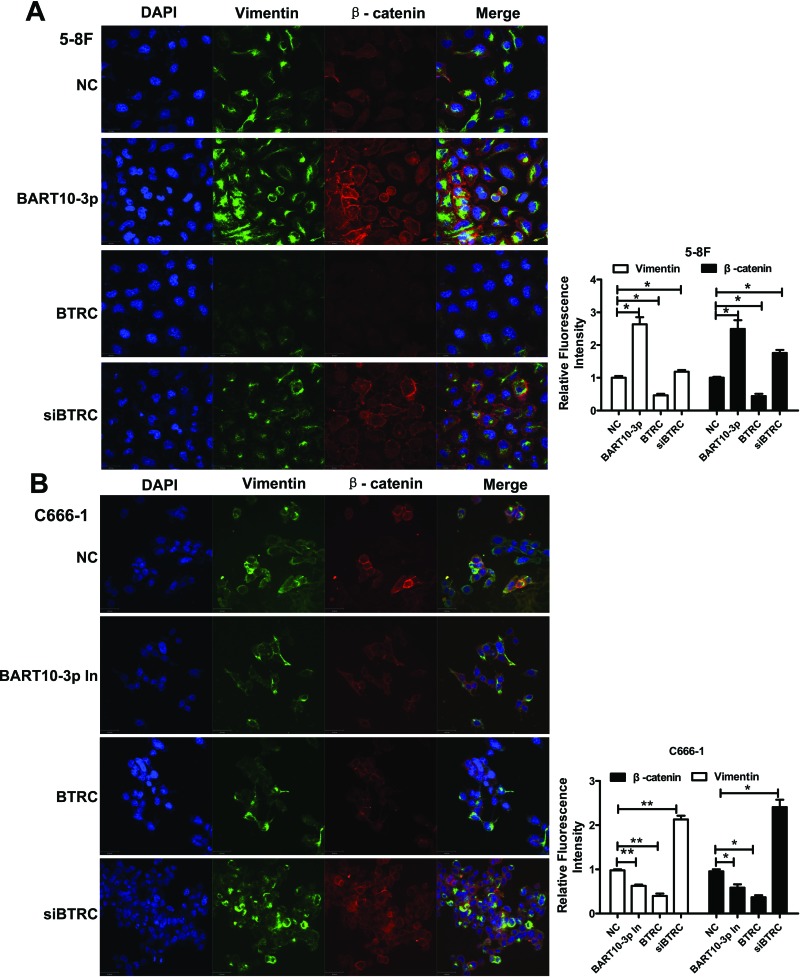
The effect of EBV-miR-BART10-3p on EMT in NPC cells was confirmed by immunofluorescence The expression levels of Vimentin and β-catenin were examined by immunofluorescence assay in 5-8F **A.** or C666-1 **B.** cells transfected with EBV-miR-BART10-3p mimics (BART-10-3p), or *BTRC* expression vector (BTRC), EBV-miR-BART10-3p inhibitors (BART-10-3p In), or *BTRC* siRNA (siBTRC). Up-regulation of Vimentin and β-catenin by both EBV-miR-BART10-3p mimics and *BTRC* siRNA, as well as down-regulation of them by ectopic *BTRC* or EBV-miR-BART10-3p inhibitors were also confirmed by this assay (NC: negative control). Five randomly selected areas were scanned and data were shown as the mean ± standard deviation (right panel, *, *p* < 0.05; **, *p* <0.01).

**Figure 7:**
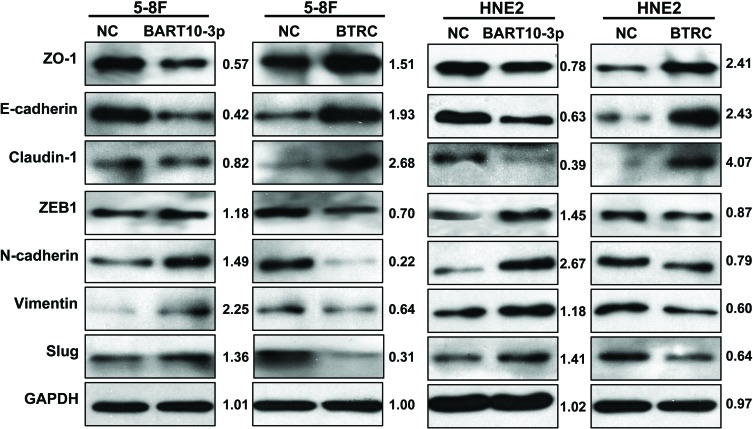
EBV-miR-BART10-3p promoted EMT through *BTRC* The expression levels of epithelial markers ZO-1, E-cadherin and claudin-1, as well as mesenchymal markers ZEB1, N-cadherin, Vimentin and Slug were examined by western blot analysis in HNE2 and 5-8F cell lines that were transfected with EBV-miR-BART10-3p mimics (BART10-3p) or *BTRC* expression vector (*BTRC*). GAPDH was used as an internal control in western blotting.

Considering that β-catenin and Snail are also two important regulators of EMT, we deemed it prudent to examine the expression of EMT-related proteins in NPC cells after transfection EBV-miR-BART10-3p mimics or inhibitor. Immunofluorescence assay results showed that overexpression of EBV-miR-BART10-3p or inhibiting *BTRC* expression by siRNA in EBV-negative cell line 5-8F could significantly increase the expression of β-catenin and the mesenchymal marker Vimentin (Figure 6A), while overexpression of *BTRC* or inhibiting endogenous EBV-miR-BART10-3p in EBV-positive cell line C666-1 could significantly decrease the expression of β-catenin and Vimentin (Figure 6B). A variety of epithelial and mesenchymal markers were validated by western blotting. Overexpression of EBV-miR-BART10-3p could significantly reduce the expression of epithelial markers, such as ZO-1, E-cadherin and Claudin-1, and increase the expression of mesenchymal markers, such as ZEB1, N-cadherin, Vimentin, and Slug. While overexpression of *BTRC* resulted in an opposite results (Figure 7). These results suggested that EBV-miR-BART10-3p was promoted the EMT and metastasis of NPC cells by targeting *BTRC* and regulating the expression of βTrCP substrates, β-catenin and Snail.

## DISCUSSION

Nasopharyngeal carcinoma (NPC) is an EBV-associated epithelial malignancy typically characterized by its early metastasis, in which lymph node metastasis to the neck and intracranial invasion is a common event [29–34]. EBV infection is tightly associated with the development of NPC, but EBV encoded miRNAs in cancer invasion and metastasis remains largely unknown. Our previous study found that a variety of cytoskeletal and adherens-related genes were potential host target genes of EBV-BART miRNAs, according to bioinformatics predictions [22]. Those findings suggested that EBV miRNAs most likely regulate tumor invasion and metastasis in NPC. Therefore, in this study, we investigated the role of EBV-miR-BART10-3p in the development of NPC, especially NPC metastasis. We found increased EBV-miR-BART10-3p expression in NPC was correlated with poor prognosis of NPC. EBV-miR-BART10-3p could promote invasion and migration of NPC cells, through a mechanism underlying the inhibition of *BTRC* expression, thereby abolishing the activity of βTrCP E3 ubiquitin ligase, leading to increased β-catenin and Snail that are two substrates of βTrCP and important roles in EMT.

EMT is a well known mechanism related to cancer metastasis. Metastasis of NPC is also a main reason for relapse and death of NPC patients. Thus, better understanding of the processes of EMT will provide a feasible therapeutic approach for the treatment of epithelial malignancy in NPC. It has been reported that EBV-miR-BART9 promoted the invasion and metastasis of NPC cells by inhibiting the expression of E-cadherin, a key membrane protein that is essential for integrity of the cell-cell junctions of epithelial cells [21]. Another study showed that EBV-miR-BART7-3p inhibited the expression of tumor suppressor PTEN, linked to the occurrence of EMT in NPC [23]. It has been demonstrated in our study that tight junction proteins such as ZO-1 and Claudin-1 were downregulated, but several mesenchymal cell related proteins like Vimentin and Slug were up-regulated in EBV-miR-BART10-3p mimics transfected NPC cells. Taken together, including the previous reports [21, 23] and our findings, we propose a hypothesis that several miRNAs located in EBV BART clusters may be collectively involved in the regulation of host genes related to EMT. It is necessary to verify this hypothesis through more in-depth studies in the future.

*BTRC* encodes βTrCP protein, a member of F-box protein family and a key component of the SCF (Skp1-Cullin1-F-box) -type ubiquitin ligase E3. The findings of the inhibitory effects of *BTRC* on cell growth and tumor formation, as well as down-regulation of *BTRC* expression in lung cancer [35] suggest that *BTRC* gene may function as a tumor suppressor to prevent the development of cancer. *BTRC* was also demonstrated to be an important factor in the process of EMT because of βTrCP-mediated ubiquitination of Snail in lung cancer, which inhibition of βTrCP resulted in the upregulation of Snail could induce EMT [36]. Another report showed that the formation of β-catenin/YWHAZ complex suppressed β-catenin from binding of βTrCP, leading to an increase in β-catenin stability and promoting EMT in lung cancer [37]. However, there are rare reports of the functions of *BTRC* gene in NPC. According to our findings, in this study, we proposed βTrCP as a novel diagnostic biomarker for NPC, because the low expression of βTrCP was a poor prognostic factor in NPC, due to its down-regulation by EBV-miR-BART10-3p.

This study also confirmed that EBV-miR-BART10-3p could induce invasion and metastasis of NPC cells by inhibiting βTrCP expression, and up-regulating the expression of its substrates β-catenin and Snail. β-catenin is a critical molecule in the WNT signaling pathway and nuclear accumulation of β-catenin participates in the transcriptional regulation of a number of genes [27]. Moreover, βTrCP also inhibits phosphorylation and ubiquitination of Snail, which is a nuclear transcription factor that promotes transcriptional activation of several downstream genes, particularly those related to EMT, thereby contributing to the occurrence of EMT [38]. βTrCP can also recognize the other specific phosphorylated substrates, such as IκBα [39], ATF4 [40], Cdc25A [41], Emil [42], Mdm2 [43], and so on. Those proteins related to βTrCP are involved in WNT signaling pathway, cell cycle [44, 45], cell invasion and metastasis [46]. The ubiquitination and proteasomal degradation of βTrCP substrates, as mentioned above, will affect cell growth [47], apoptosis [48–51], and tumorigenesis [52]. It is still not clear whether EBV-miR-BART10-3p participates in the process of carcinogenesis and development of NPC through the regulation of other βTrCP substrates via down-regulation of *BTRC* expression. It will be an interesting direction of our future studies.

**Figure 8:**
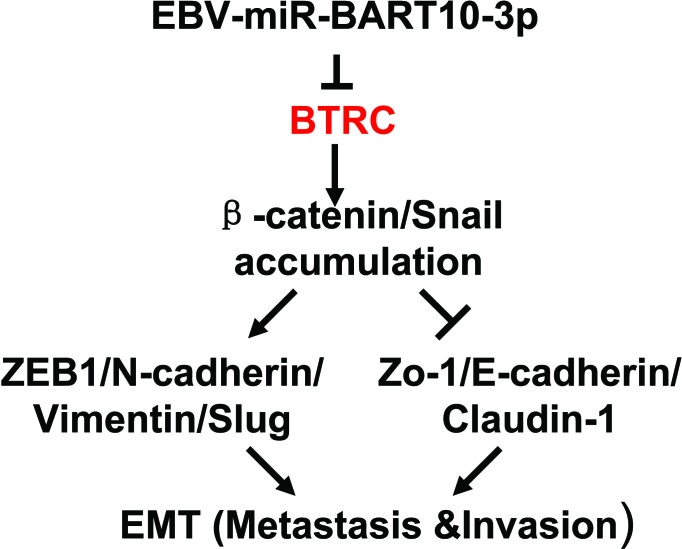
Graphical representation illustrated the role of EBV-miR-BART10-3p mediated pathway in the EMT of NPC

In conclusion, our study revealed that overexpressed EBV-miR-BART10-3p could promote invasion and migration of NPC cells, through inhibition of its target *BTRC* expression, thereby inhibiting the ubiquitination of βTrCP downstream substrates β-catenin and Snail, leading to the regulation of many EMT related molecules, such as downregulated expression of E-cadherin, tight junction protein ZO-1 and Claudin-1, as well as upregulated E-box binding zinc finger protein ZEB1 and N-cadherin (Figure 8). This study presented a new mechanism of EBV infection in NPC carcinogenesis. Meanwhile, our findings suggested that EBV-miR-BART10-3p might be a novel biomarker for NPC diagnosis and prognosis as well as a potential therapeutic target for NPC patients.

## MATERIALS AND METHODS

### Clinical specimens

There ware 9 fresh non-tumor nasopharyngeal epithelial tissue samples 28 and NPC biopsies were collected for Real-time PCR, and 10 paraffin-embedded non-tumor nasopharyngeal epithelial tissue samples and 106 NPC samples were used for *in situ* hybridization or immunohistochemistry to measure EBV-miR-BART10-3p and *BTRC* expression. All tissue samples were collected from newly diagnosed NPC patients at the Affiliated Cancer Hospital of Central South University (Changsha, China), which was approved by the hospital Research Ethics Board. The signed informed consent was also obtained from each participant before they were enrolled in the study. The diagnoses of all specimens were confirmed by histopathological examination. All patients recruited in our study had received routine radiotherapy. Clinicopathological data were collected from patient medical records and are reported in Supplemental Tables S1.

### *In situ* hybridization and immunohistochemistry

The probes for *in situ* hybridization were synthesized and labeled with DIG-dUTP at the 3′ end from Exiqon (Exiqon, Vedbaek Denmark) and the procedure to detect EBV-miR-BART10-3p expression was as previously described with a few modifications [53–55]. Briefly, the slides were treated with pepsin diluted in 3% citric acid for 15 min and prefixed with 4% paraformaldehyde for 10 min after deparaffin, and then prehybridization and hybridized with 50 nM DIG-labeled EBV-miR-BART10-3p probe at 53°C overnight.

For immunohistochemistry (IHC), the slides were incubated in antigen retrieval buffer (0.01M citrate buffer) for 30 min after deparaffin. Then the slides were incubated with primary antibody (βTrCP, Cell Signaling, Danvers, MA) at 4°C overnight. After washing with PBS for three times, the sections were incubated with polymerized HRP and anti-rabbit IgG for 30 min.

To evaluate the amount of ISH or IHC positive cells, a semi-quantitative scoring criterion was used to estimate both staining intensity and positive areas. The scores corresponding to the overall distribution of EBV-miR-BART10-3p signal and βTrCP immunoreactivity were averaged across the different tumor plugs in each case. Slides were recorded as noninformative, if the tissue was lost in processing; there was no recognizable tumor in the slide; or there were extensive staining artifacts (eg, inappropriate staining of collagen or tissue edges and tissue creases in a specimen with minimal tissue retained). The scoring was graded as 0 (negative), 1 (<10% positive), 2 (10%-50% positive), or 3 (>50% positive) in accordance with the staining proportion and intensity. The final scores were regarded as low expression (0-1) and high expression (2-3) [56]. All sections were independently scored by two pathologists who were blinded to the clinicopathological features.

### Cell lines and constructs for transfection

NPC cell lines were maintained in RPMI-1640 medium, including EBV negative cell lines HNE2, 5-8F, and positive cell line C666-1. Synthetic EBV-miR-BART10-3p mimics and inhibitors were products of Qiagen Company (Qiagen, Hilden, Germany). EBV-miR-BART10-3p inhibitor was a chemically synthesized, single-stranded, modified RNA molecule that can specifically inhibit endogenous target miRNA, when cells were transfected with this inhibitor. Full-length cDNA of *BTRC* was amplified by PCR and constructed by inserting a PCR product into pIRESneo3 vector. *BTRC* siRNAs (5′-CCCAGGGACUGGCGCACUCdTdT-3′) were obtained from Genepharma (Shanghai, China). The luciferase reporter was established by inserting synthetic oligonucleotides containing either wild-type EBV-miR-BART10-3p binding site in *BTRC* 3′-UTR (*BTRC*-WT) or the one with mutant-binding site (*BTRC*-Mutant) into pmiR-Report luciferase vector (Ambion, Austin, TX,). The sequences of the synthetic oligonucleotides were as follows: (1) 3′-UTR of *BTRC* containing wild-type binding site of EBV-miR-BART10-3p: 5′ - CTAGTCCA ACCAGCACAGCTGGCGCTCTTAGCTCCTGATTGG TTGTGTGTTTTATTAAA-3′ and 5′ - AG CTTTTAATAAAACACACAACCAATCAGGAGCTAA GAGCGCCAGCTGTGCTGGTTGGA-3′; (2) 3′-UTR of mutant *BTRC*, in which the seed sequence of EBV-miR-BART10-3p (the binding site) was changed: 5′ - CTAGTCCAACCAGCACAGCTGGCGC TCTTAGCTCCTGATTAAAGTACGGTTTTATTAAA-3′ and 5′ - AGCTTTTAATAAAA CCGTACTTTAATCAGGAGCTAAGAGCGCCAGCTGT GCTGGTTGGA-3′. Transfection of plasmids and miRNAs was performed with Attractene or HiPerFect transfection reagents (Qiagen) as recommended.

### Luciferase assay

Cells were plated into each well of a 24-well plate and then co-transfected with synthetic EBV-miR-BART10-3p mimics and luciferase reporter plasmids (either *BTRC*-WT or *BTRC*-Mutant), also along with pRL-TK renilla luciferase vector (Promega, Madison, WI). Luciferase activity was measured using the Dual-Luciferase^®^ Reporter Assay System (Promega). All experiments were performed three times.

### Northern blots and quantitative real-time PCR

Total RNA was isolated with TRIzol reagent (Invitrogen, Carlsbad, CA), according to manufacturer's protocol. For Northern blots analysis of EBV-miR-BART10-3p miRNA expression, miRNA Northern Blot Assay Kit (Signosis, Santa Clara, CA) was performed using 5 μg total RNA according to the manufacturer's protocol. Expression of EBV-miR-BART10-3p was detected with a biotin-labeled probe, containing full-length antisense DNA oligonucleotides of EBV-miR-BART10-3p. The U6 RNA was used as a control. For real-time PCR, cDNA was synthesized using miScript system (Qiagen), following manufacturer's instructions. The expression level of EBV-miR-BART10-3p was measured by Qiagen miRNA primer assays (Qiagen) using the miScript SYBR^®^ Green real-time PCR Kit (Qiagen), in compliance with manufacturer's instructions. Data was normalized to the expression level of small nuclear RNA RNU6B (U6 snRNA). Real-time PCR for *BTRC* was carried out using a SYBR green real-time PCR kit (TaKaRa, Tokyo, Japan). Data were normalized to the expression level of GAPDH and further normalized to the negative control, unless otherwise indicated. The primers used for PCR were *BTRC* (forward) 5′-CCCCTTCTCGAACATACACCT-3′, and (reverse) 5′-AGTCTCAAAGCCCTGCTCCT-3′ as well as *GAPDH* (forward) 5′-AACGGATTTGGTCGTATTGG-3′ and (reverse) 5′-TTGATTTTGGAGGGATCTCG-3′. The fold changes were calculated by relative quantification (2 ^−ΔΔCt^) method. All reactions were run in triplicate and repeated in three independent experiments.

### Western blot analysis

The protein was extracted using Radio-Immunoprecipitation Assay Buffer (RIPA buffer, Santa Cruz, CA) and the protein concentration was determined using the BCA^TM^ Protein Assay Kit (Pierced, Grand Island, NY). Samples were separated by electrophoresis on 10-12% sodium dodecyl sulfate (SDS) polyacrylamide gels, and the separated proteins were transferred to a polyvinylidene fluoride (PVDF) membrane (Millipore, Billerica, MA). To assess the protein expression, the blots were incubated with the following primary antibodies at 4°C overnight: rabbit antibodies against βTrCP, ZO-1, E-cadherin, ZEB1, N-cadherin, Vimentin, and Slug (Cell Signaling Technology), as well as mouse antibodies against Snail (Cell Signaling Technology), and β-catenin (BD Biosciences, New Jersey). After washing, the blots were incubated with horseradish peroxidase-conjugated anti-rabbit secondary antibodies (Cell Signaling Technology) at a dilution of 1:2000 for 1 h at room temperature. Blots were visualized by exposure to X-ray film, through an enhanced chemiluminescence detection system (Millipore). GAPDH (Cell Signaling Technology) served as an endogenous control for equal loading.

### Wound healing and transwell assay

For wound healing assay, when the cells were grown to 90% confluence after transfection, a straight scratch in the cell monolayer was created by a 10 μL pipette tip. Images of the scratched area (wound) were taken at the time point of 0h, 24 h, and 48 h under a microscope. For transwell assay, cells were seeded in the chamber (8 μm pores; Corning, NY) coated with Matrigel (BD Biosciences). Then the chamber was placed into a 24-well plate and incubated at 37°C for 12-24 hours. Finally, the chambers were fixed with 4% paraformaldehyde for 10 min and invasive cells were examined by crystal violet staining [53].

### Immunofluorescence

Cells were seeded on coverslips in a 6-well plate and fixed in 4% paraformaldehyde for 20 min, followed by permeabilization of cell membranes with 0.5% Triton X-100 for 3 min and blocked in phosphate-buffered saline (PBS) containing fetal bovine serum (FBS) for 30 min after transfection. Then the cells were incubated with primary antibodies, including rabbit anti-Vimentin (1:50, Cell Signaling Technology) and mouse anti-β-catenin (1:100, BD Biosciences) at 4°C overnight. Next day, after washes, secondary antibodies either FITC-conjugated sheep anti-rabbit IgG 1:2000 or Cy3-conjugated sheep anti-mouse IgG (diluted 1:2000 in PBS) was used (Boster; Wuhan, China) for 1 hour incubation. Meanwhile, DAPI (4′,6-diamidino-2-phenylindole) was also used to stain nuclei in the cells.

### Statistical analysis

Statistical analysis was performed using software of SPSS16.0 (SPSS, Chicago, IL) and Graph Pad Prism 5 (GraphPad, La Jolla, CA). Student's t-tests were used to evaluate significant differences between any two groups of data. One way ANOVA was used when there are more than two groups. Disease-free survival (DFS) was defined as the time relapsed between the diagnosis and the date of first treatment failure. The OS and DFS estimates over time were calculated using the Kaplan-Meier method, and the differences were compared using the log-rank test. The results of the analysis were considered significant in a log-rank test if *p* < 0.05. All data are represented as means ± standard deviation. Differences were considered significant if *p* < 0.05.

## SUPPLEMENTARY TABLE


